# Gene functional networks and autism spectrum characteristics in young people with intellectual disability: a dimensional phenotyping study

**DOI:** 10.1186/s13229-020-00403-9

**Published:** 2020-12-11

**Authors:** Diandra Brkić, Elise Ng-Cordell, Sinéad O’Brien, Gaia Scerif, Duncan Astle, Kate Baker

**Affiliations:** 1 MRC Cognition and Brain Sciences Unit, University of Cambridge, 15 Chaucer Road, Cambridge, CB2 7EF UK; 2 Department of Experimental Psychology, University of Oxford, Anna Watts Building, Radcliffe Observatory Quarter, Woodstock Road, Oxford, OX2 6GG UK

**Keywords:** Autism dimensions, Intellectual disability, Genetics, Hyperactivity, Anxiety

## Abstract

**Background:**

The relationships between specific genetic aetiology and phenotype in neurodevelopmental disorders are complex and hotly contested. Genes associated with intellectual disability (ID) can be grouped into networks according to gene function. This study explored whether individuals with ID show differences in autism spectrum characteristics (ASC), depending on the functional network membership of their rare, pathogenic de novo genetic variants.

**Methods:**

Children and young people with ID of known genetic origin were allocated to two broad functional network groups: synaptic physiology (*n* = 29) or chromatin regulation (*n* = 23). We applied principle components analysis to the Social Responsiveness Scale to map the structure of ASC in this population and identified three components—Inflexibility, Social Understanding and Social Motivation. We then used Akaike information criterion to test the best fitting models for predicting ASC components, including demographic factors (age, gender), non-ASC behavioural factors (global adaptive function, anxiety, hyperactivity, inattention), and gene functional networks.

**Results:**

We found that, when other factors are accounted for, the chromatin regulation group showed higher levels of Inflexibility. We also observed contrasting predictors of ASC within each network group. Within the chromatin regulation group, Social Understanding was associated with inattention, and Social Motivation was predicted by hyperactivity. Within the synaptic group, Social Understanding was associated with hyperactivity, and Social Motivation was linked to anxiety.

**Limitations:**

Functional network definitions were manually curated based on multiple sources of evidence, but a data-driven approach to classification may be more robust. Sample sizes for rare genetic diagnoses remain small, mitigated by our network-based approach to group comparisons. This is a cross-sectional study across a wide age range, and longitudinal data within focused age groups will be informative of developmental trajectories across network groups.

**Conclusion:**

We report that gene functional networks can predict Inflexibility, but not other ASC dimensions. Contrasting behavioural associations within each group suggest network-specific developmental pathways from genomic variation to autism. Simple classification of neurodevelopmental disorder genes as high risk or low risk for autism is unlikely to be valid or useful.

## Background

Intellectual disability (ID, defined as IQ < 70 plus impaired adaptive function) and autism spectrum disorder (ASD, defined as persistent deficits in social communication and social interaction plus restricted, repetitive behaviours, interests, or activities) frequently co-occur, but are not synonymous [1]. The potential for dual ID-ASD diagnosis recognises that autism characteristics vary within the ID population and are not an inevitable consequence of low cognitive ability and adaptive impairments. Understanding autism within the ID population is important, because autism predicts the complexity of educational, occupational, and social support needs [2] and influences the well-being of family carers [3]. One factor which can influence behavioural phenotypes, including autism, is the aetiology of each individual’s ID. At least 60% of individuals with severe ID have an underlying genetic diagnosis, which can now be readily diagnosed [4], opening up new opportunities to understand the role of aetiology in shaping the cognitive and interpersonal development of individuals with ID. Moreover, identifying factors which predict the association between ID and autism could highlight causal influences and mechanisms which are critical mediators of social development.

However, the relationship between genetic diagnoses and autism is hotly contested. Some large cohort studies have presented evidence for “autism-predominant” neurodevelopmental disorder genes [5, 6], implying that autism is a unitary and categorical phenotype, and risk of this phenotype can be strongly influenced by the presence of a single specific causal factor. However, others argue strongly against this classification on both theoretical and empirical grounds [7]. An alternative proposal is that autism is a multi-faceted construct, and that specific causal factors could influence aspects of the construct selectively, to variable degrees, in complex interaction with other influences.

To resolve this debate, systematic phenotyping is required to determine whether and how the genetic cause of ID predicts autism spectrum characteristics (ASC). However, addressing this question on a gene-by-gene basis holds several methodological challenges. Firstly, the rarity of each genetic disorder means that knowledge of phenotypic spectra can be skewed by small case numbers, not taking into account the expected variation in phenotypes within small groups, and rarely comparing across aetiologies associated with similar levels of ID severity. Secondly, cohort studies have typically relied on primary ascertainment diagnosis, or retrospective coding from medical notes, rather than acquiring standardised data. Thirdly, existing studies mainly focus on the presence or absence of categorical ASD diagnosis, rather than recognising that the characteristics contributing to ASD are diverse and vary along continuous dimensions such as social communication and repetitive behaviours [8, 9]. Previous studies of well-known syndromes associated with ASD, for example Fragile X Syndrome and Tuberous Sclerosis Complex, have highlighted considerable variation in atypical social behaviours contributing to ASD and different predictors of ASD within each syndrome group [10, 11]. In essence, to understand autism in the context of ID-associated genetic disorders it is necessary to move beyond categorical diagnosis to investigate diverse aspects of social behaviour plus other aspects of ID such as non-social attention and affective regulation.

In the current study, we apply novel strategies to explore the relationships between genetic aetiology and ASC in young people with ID. Our first strategy is to reduce ascertainment bias by recruiting individuals after genetic diagnosis, irrespective of primary indication for genetic testing. Our second strategy is to collect standardised carer-report phenotyping assessments, appropriate for individuals with ID. Thirdly, we take a data-driven approach to analyses, by mapping the component structure of ASCs at single item level, then modelling predictors within the sample. Fourthly, we adopt a functional network phenotyping approach, meaning that we group participants according to known molecular and cellular functions of genetic variants, to detect convergent influences on behavioural outcomes, and provide insights into cognitive and neural mechanisms linking genetic cause to behavioural outcome [12]. The current study compares two functional networks—a narrowly defined group of chromatin structural modifiers (components and regulators of the SWI/SNF chromatin remodelling complex) and a broader group encompassing direct and indirect modifiers of synaptic physiology. Chromatin modelling is essential for the establishment and maintenance of gene expression profiles to support neuronal differentiation, structural brain organisation, and flexibility of neuronal circuitry for learning [13, 14]. Synaptic transmission, its upstream regulation, and downstream signalling are fundamental to dynamic neurophysiological processes supporting perception, memory, and action [15]. In this exploratory study, we set out to establish (1) whether the distribution of autism characteristics within this sample was categorical, unidimensional, or multidimensional, (2) whether functional network group membership influenced likelihood of autism characteristics, and (3) whether the predictors of autism characteristics were the same or different between functional network groups, reflecting shared or distinct underlying mechanisms.

## Methods

### Recruitment

Participants had been clinically identified as having neurodevelopmental impairments (developmental delay, intellectual disability, or behavioural difficulties; in isolation or in association with other phenotypes) and referred for diagnostic genetic testing via clinical or research pathways. Genetic testing had been carried out via whole exome sequencing or gene panel testing. A pathogenic or likely pathogenic sequence variant had been identified in any gene associated with a neurodevelopmental phenotype according to https://www.ebi.ac.uk/gene2phenotype. The recruitment strategy did not involve selecting for a specific list of genetic diagnoses or gene functions. Genetic disorders associated with multi-system phenotypes (congenital abnormalities or physical health difficulties) were not excluded. Metabolic and mitochondrial disorders were excluded. Variants identified via research pathways had been validated in a clinical laboratory. Each participant’s variant had been evaluated by local clinical geneticist as being a causal or contributory factor for the individuals’ neurodevelopmental presentation, and genetic counselling completed. Information about the current study was then provided to participants’ families via regional genetics services, other clinical services, other research projects, family support groups, and via the project website. Parents of children under 16 gave written informed consent on behalf of their child. For participants with ID over the age of 16 lacking capacity to consent, a consultee was appointed.

### Group definitions

After recruitment to this study, every participant’s genetic diagnosis was evaluated in order to allocate participants to functional network groups (FNGs). Information about each gene was manually curated based on consistent documentation of biochemical function, synaptic proteomics, GeneOntology (biological class), and PubMed searching (Additional file 1: Table S1). Information was curated by a single individual (KB), and consensus reached amongst the authorship on FNG definitions and gene allocations. FNGs comprised (1) genes involved in chromatin structural regulation (“chromatin” group), and (2) genes involved in synaptic transmission, synapse-associated cytoskeleton or post-synaptic intracellular signalling (“synaptic” group). Participants in the study had variants in 15 different genes: 23 participants had variants in one of five chromatin genes (*ARID1B, SETD5, EHMT1, KAT6B, SMARCA2*), and 29 participants with variants in one of ten synaptic genes (*CASK, CTNNB1, DDX3X, DLG3, DYRK1A, PAK3, SHANK3, STXBP1, TRIO, ZDHHC9*) (Additional file 1: Table S2).

**Table 1:** Demographic and behavioural characteristics

	Chromatin (*N* = 23)		Synaptic (*N* = 29)		*t*-test
	Mean (SD)	Range	Mean (SD)	Range	Chromatin–synaptic
Gender	11F:12M	–	19F:10M	–	–
Age	12.93 (5.14)	5 to 25	15.38 (5.23)	7 to 26	*t*(50) = − 1.69 *p* = 0.097 *d* = 0.47
Vineland composite	64.96 (11.90)	41 to 96	49.59 (13.97)	20 to 79	*t*(50) = 4.20 *p* < 0.001 *d* = 1.18
SRS total (T)	76.30 (12.73)	53 to 96	75.52 (11.30)	49 to 98	*t*(50) = 0.24 *p* = 0.814 *d* = 0.06
SRS raw score > 60 (clinical significance)	19/23 (82.60%)	–	26/29 (89.66%)	–	–
CPRS inattention (T)^a^	79.71 (11.49)	60 to 90	80.50 (10.99)	55 to 90	*t*(43) = − 0.23 *p* = 0.816 *d* = 0.07
CPRS hyperactivity (T)^a^	69.05 (14.55)	40 to 90	76.92 (15.03)	42 to 90	*t*(43) = − 1.78 *p* = 0.082 *d* = 0.53
DBC total (percentile)	61.65 (28.59)	2 to 100	67.93 (26.49)	18 to 98	*t*(50) = − 0.82 *p* = 0.416 *d* = 0.23
DBC anxiety (percentile)	55.39 (28.09)	10 to 98	56.55 (25.51)	10 to 98	*t*(50) = − 0.16 *p* = 0.877 *d* = 0.04
Factor 1 (Inflexibility)^b^	0.23 (1.06)	− 1.52 to 2.20	− 0.18 (0.93)	− 1.89 to 1.58	*t*(50) = − 1.49 *p* = 0.143 *d* = 0.41
Factor 2 (Social understanding)^b^	− 0.17 (1.13)	− 2.39 to 1.90	0.13 (0.88)	− 2.13 to 1.45	*t*(50) = − 1.09 *p* = 0.280 *d* = 0.30
Factor 3 (Social motivation)^b^	− 0.03 (1.11)	− 1.67 to 2.03	0.02 (0.92)	− 1.42 to 1.71	*t*(50) = − 0.167 *p* = 0.868 *d* = 0.05

^a^45 of 52 participants (21 in the chromatin group and 24 in the synaptic group) completed the CPRS

^b^Factor scores were transformed to z-scores, with a mean of 0 and standard deviation of 1

**Table 2:** AIC models for autism spectrum characteristics, within whole sample

Component	Models	N variables	AIC weight	AIC_c_	ΔAIC	Residual deviance
Inflexibility	Anxiety + FNG + Hyperactivity + Vineland	4	0.221	100.21	0	17.81
	Anxiety + FNG + Hyperactivity	3	0.202	100.39	0.18	18.98
	Anxiety + FNG + Hyperactivity + (FNG × Hyperactivity)	5	0.175	100.68	0.466	18
Social understanding	Anxiety + FNG + Hyperactivity + Inattention + Vineland + FNG × Hyperactivity + FNG × Inattention	9	0.174	114.93	0	20.26
	FNG + Hyperactivity + Inattention + Vineland + FNG × Hyperactivity + FNG × Inattention	8	0.133	115.47	0.537	21.98
	Anxiety + FNG + Gender + Hyperactivity + Inattention + Vineland + FNG × Hyperactivity + FNG × Inattention	10	0.131	115.49	0.564	19.05
Social motivation	Hyperactivity + Inattention	2	0.34	127.12	0	36.37
	Hyperactivity	1	0.3328	127.19	0.071	38.43
	Age + Hyperactivity	2	0.199	128.19	1.07	37.24

Summary of the best set of three similarly supported models, for each ASC dimension. *N* variables = number of parameters for each model, AIC weight = is the probability of each model being the best model, or relative evidence for each model. These estimates are computed by normalising model likelihoods. AIC_c_ = AIC criterion of model selection, corrected for smaller sample size, ΔAIC = AIC difference between the best fitting model (equal to zero) and the second best model. Residual deviance = distance between the data and the model

### Questionnaire and interview measures

Parents or carers completed the Vineland Adaptive Behaviour Scales, Second Edition, Survey Interview Form (Vineland; 16), Social Responsiveness Scale, Second edition (SRS; 17), Developmental Behaviour Checklist (DBC; 18), and Conners Parent Rating Scales (CPRS; 19).

### Data analysis

We first addressed whether autism in this study population is best conceptualised as a unidimensional or multidimensional construct, via principal components analysis (PCA) of SRS items. In line with previous studies [8], component solution was selected on: (1) scree plots/percentage of variance explained, and (2) conceptual interpretability. We applied orthogonal rotation (varimax with Kaiser normalization) to identify potentially diverging dimensions and underlying mechanisms. While we would expect that SRS total and dimension scores were normally distributed, simple group comparisons were made via independent samples t tests.

To identify predictors of ASC dimensions we applied Akaike’s information criterion (AIC) modelling, corrected for small sample sizes (AICc). Information criteria modelling approaches allow inference from more than one model, controlling for over-dispersion and taking into account goodness of fit [20], when the true model is too complex to be estimated parametrically [21, 22]. AIC models included all participants with complete questionnaire and interview data (*N* = 45). A consistent set of potential predictors were included across all analyses: age, gender, global ability (Vineland Adaptive Behavior Composite), inattention (CPRS inattention subscale), hyperactivity (CPRS hyperactivity subscale), and anxiety (DBC anxiety subscale). Analyses comprised two steps, (1) a model selection step, geared to identify the best fitting models based on AICc values, with the most parsimonious models (i.e. lowest AIC value) favoured; (2) a multi-modal inference step, geared to infer the weight of individual predictors relative to the others, and the associated confidence intervals. Both of these steps are described in more detail below. Interaction terms were included within the models, to assess whether predictors were the same or different between groups. Analyses were performed using *glmulti* package in R [23].

*Model selection* For each ASC component resulting from the PCA, we included the same set of variables: age, gender, global adaptive ability, FNG, and non-ASD behavioural traits (inattention, hyperactivity, and anxiety). Additionally, we explored interactions between genetic diagnosis (FNG) and predictors, to investigate shared vs distinctive associations. To do this we included in the model selection paradigm interaction terms between FNG and (1) inattention, (2) hyperactivity, and (3) anxiety. AICc values were compared, with the most parsimonious models (i.e. lowest AIC value) favoured. The selection criteria for best fitting models were based on ΔAIC or difference in AIC from between a model i and the first-ranked model [34]. Generally, models with ΔAIC < 2 provide a substantially good fit to the data [20].

*Multi-model inference* In order to provide stable inference and parameter estimation, for each of the behavioural characteristics, we averaged across the top ranked models (ΔAIC < 2) and computed the single coefficients’ importance. The relative importance of the predictors, or coefficients, measures the relative likelihood that each predictor is part of the best model (Symonds 2011). This is estimated by summing the Akaike weights (ωAIC) across all the models in the candidate set. In short, the larger the weight is, the more important the variable or predictor, relative to the others. The arbitrary threshold of 0.8 was applied as a consistent cut-off for identifying the most relevant predictors (i.e. estimates that appear in more than 80% of models). This procedure allows us to look at effects of closely related models, by measuring confidence intervals and thus reducing model uncertainty [34].

## Results

### Sample characteristics

Demographics and descriptive data are displayed in Table 1. Fifty-two individuals (30 females) took part in the study. Groups were well-matched in gender and age. The chromatin group had higher levels of global adaptive ability than the synaptic group. The parents of 8 participants reported that their child had received a clinical diagnosis of ASD, pervasive developmental disorder not otherwise specified, or atypical autism, evenly distributed across FNGs. Groups did not differ in total SRS score, or % above cut-off for possible clinical diagnosis of ASD (*Χ*^2^ = 0.547, *p* = 0.46). Groups also did not differ significantly in non-ASC emotional and behavioural scores (DBC total, DBC anxiety subscale, Conners-3 inattention, and hyperactivity subscales).

### Mapping the structure of autistic behaviours in ID

We conducted PCA to map the dimensional structure of SRS-2 responses within this study population. First, PCA was run on all 65 items of the SRS-2. On visual inspection of the scree plot, there was a steep drop-off in the variance explained between three (37.1% variance explained) and four (41.4% variance explained) components (Additional file 1: Figure S1). Solutions containing one, two, three, four, and five components were examined conceptually. Again, a three-component solution appeared to be optimal: with four- and five-component solutions, similar items were split into overlapping components, whereas with one- and two-component solutions, many items within the components were not aligned. Conceptually, our solution bears similarity to the model proposed by Nelson et al. [9], who explored the factor structure of SRS teacher-reported scores in children with autism and cognitive impairments. Based on these combined findings, a three-component solution was selected. In a second step, items with communalities < 0.4 (35 items) were excluded to maximise the overall communalities. The remaining 30 items were subjected to a second PCA. The full rotated component matrix for the three-component solution is displayed in Additional file 1: Table S3. Items showing cross-loading were included. In the final model, the KMO value was 0.597, and Bartlett’s test was significant (*p* < 0.001), i.e. sampling adequacy and data structure were appropriate for PCA with this reduced number of items. The model accounted for 51.87% of variance in item scores. Component 1 (Inflexibility) accounted for 22.62% of the variance in SRS item scores and includes items related to behavioural and cognitive flexibility, as well as ritualistic or compulsive behaviour (e.g. difficulty with changes to routine, fixated patterns of thought, or sensory sensitivity). Component 2 (Social Understanding) accounted for 19.69% of variance and pertains to social awareness and cognition (e.g. knowing when invading others’ personal space, offering comfort to others when they are sad, or understanding cause and effect relations between events). Component 3 (Social Motivation) accounted for 9.56% of variance and includes items related to disinhibition or withdrawal in social situations (e.g. avoiding starting interactions with others, avoiding emotional closeness with others, or having poor self-confidence in social settings). Mean component scores did not differ significantly between groups (Table 1). As a secondary analysis, we applied oblique rotation (Promax), and findings converged with primary analyses (Additional file 1: Table S4). In summary, we found evidence for three ASC dimensions within this dataset and progressed to explore the predictors of these dimensions separately.

### Whole sample predictors of ASC components

**Fig. 1:**
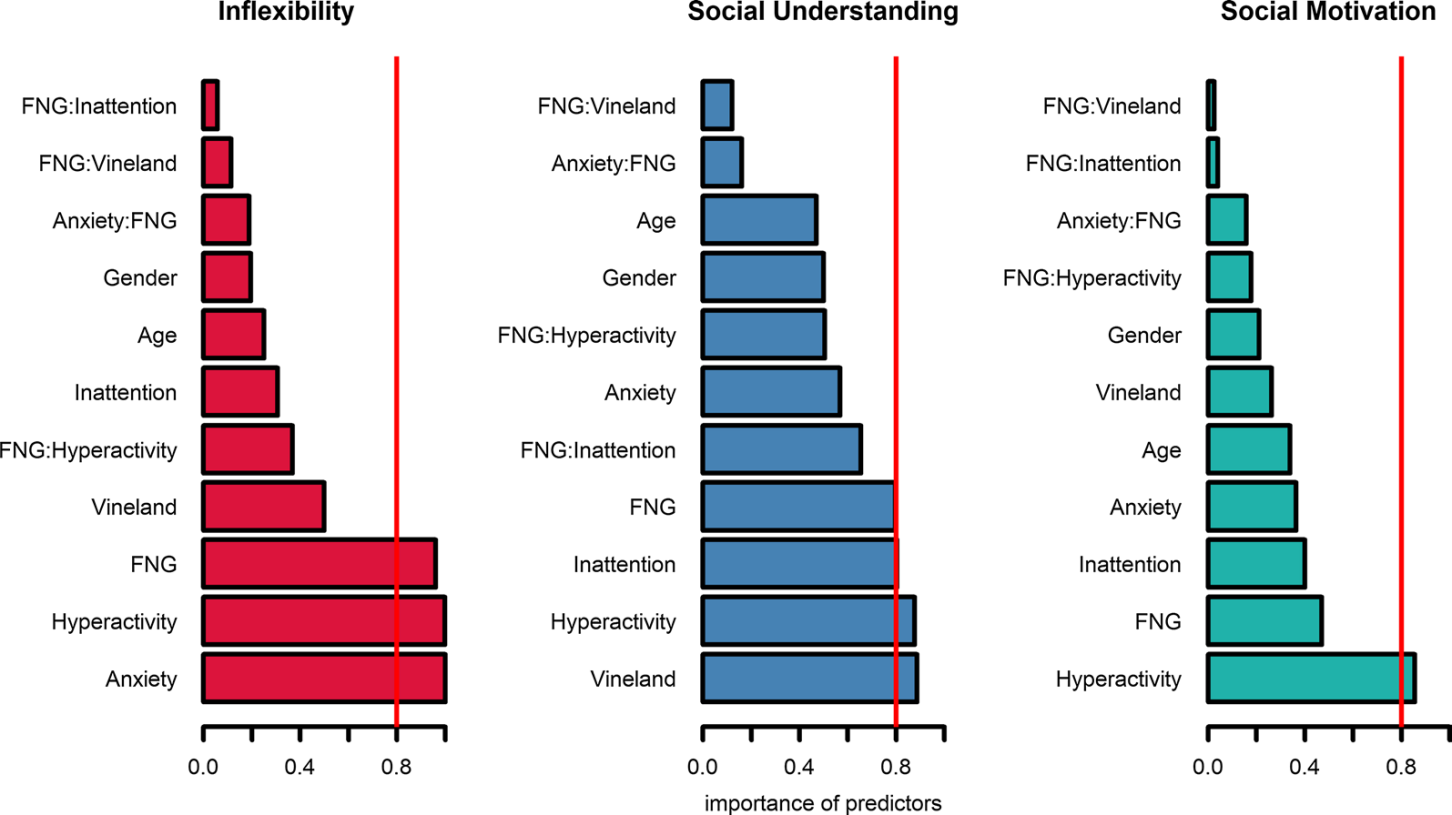
Relative importance of predictors for each ASC component, averaged across the set of candidate models. The importance of predictors was calculated by summing the Akaike weights over the subset of candidate models in which the predictor is present. The arbitrary threshold of 0.8 was applied as cut-off for identifying the most relevant predictors (i.e. estimates that appear in more than 80% of models). FNG = Functional Network Group (synaptic or chromatin)

**Fig. 2:**
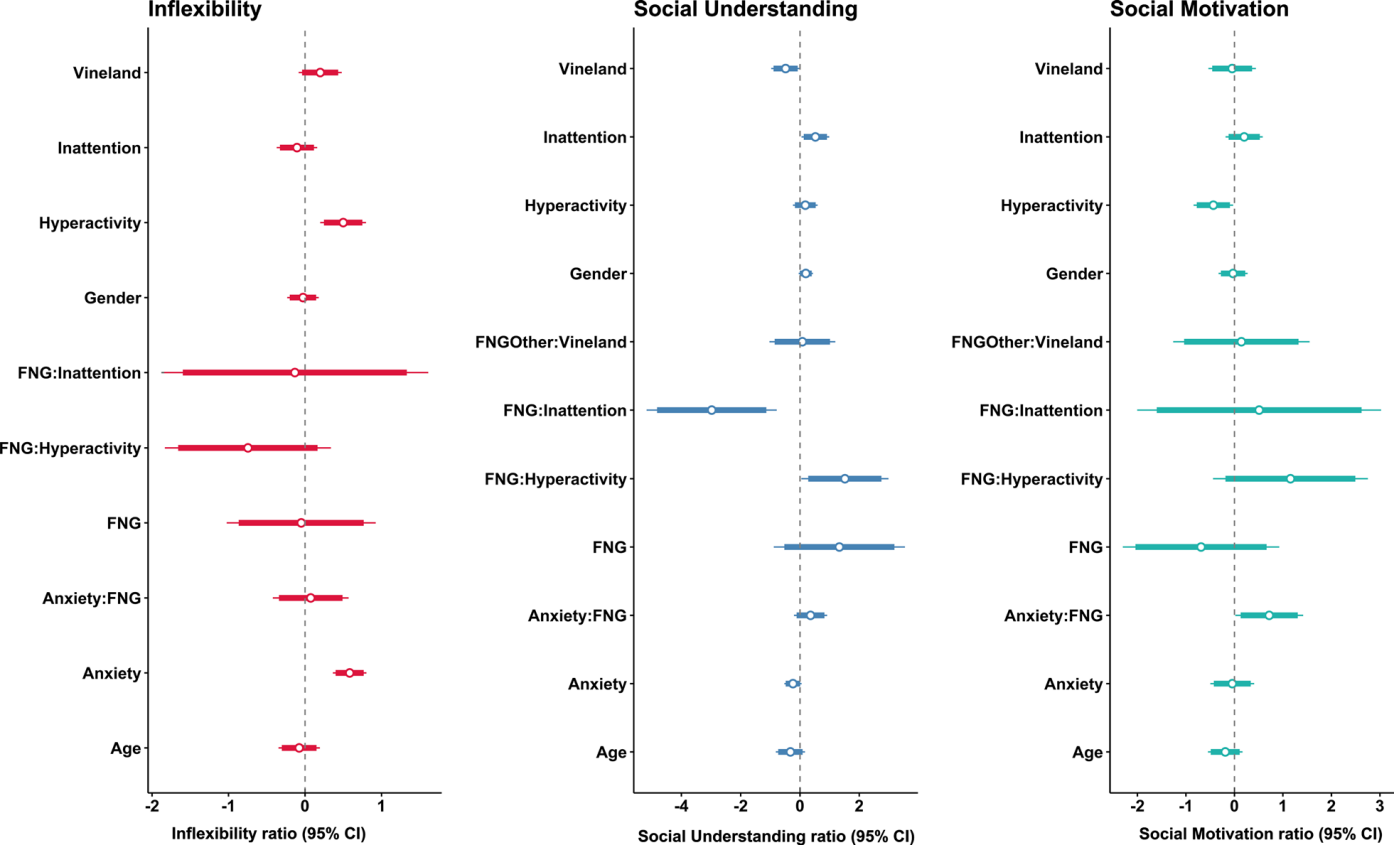
Effect size plots of estimated coefficients for each ASC. This figure illustrates model averaged coefficients and their effect sizes. The white dot is the beta coefficient value for each estimate, the thick lines are the confidence intervals (95% CI), and the lighter lines are the standard errors (SE). FNG = Functional Network Group refers to the synaptic–chromatin grouping

We then applied AIC_c_ model selection to identify predictors of each ASC dimension within the whole sample. In particular, we employed multi-model inference to compute weighted estimates of the predictive values of each of the parameters considered [20]. For each ASC component, three top-ranked AIC models with goodness of fit indices (AIC weights, deviance, and ΔAIC) are provided in Table 2. For Inflexibility, the top-ranked model had an AIC weight of 0.221 or 22% of probability of being the best model. For Social Understanding, the top-ranked model AIC weight was 0.174. For Social Motivation, the top-ranked model AIC weight was 0.34. There were multiple models competing for the top rank (ΔAIC < 2; see Table 2 and Additional file 1: Table S6). To reduce model uncertainty, model averaging and parameter estimation were calculated for each ASC dimension (Fig. 1). This indicated that the most important predictors of Inflexibility were anxiety, hyperactivity, and genetic group (FNG). Higher Inflexibility was associated with higher levels of hyperactivity and anxiety and being in the chromatin group. For Social Understanding, likely predictors of impairment were lower global adaptive ability and elevated hyperactivity. For Social Motivation, only hyperactivity was predictive across the sample, with lower levels of hyperactivity associated with social withdrawal. For effect sizes of each coefficient and predictor, see Fig. 2. In summary, multi-model inference highlighted hyperactivity as a likely predictive factor across all dimensions, anxiety, and global adaptive function as predictors of single dimensions and FNG (chromatinopathy > synaptopathy) as a predictor of Inflexibility.

### Within-group predictors of ASC components

**Fig. 3:**
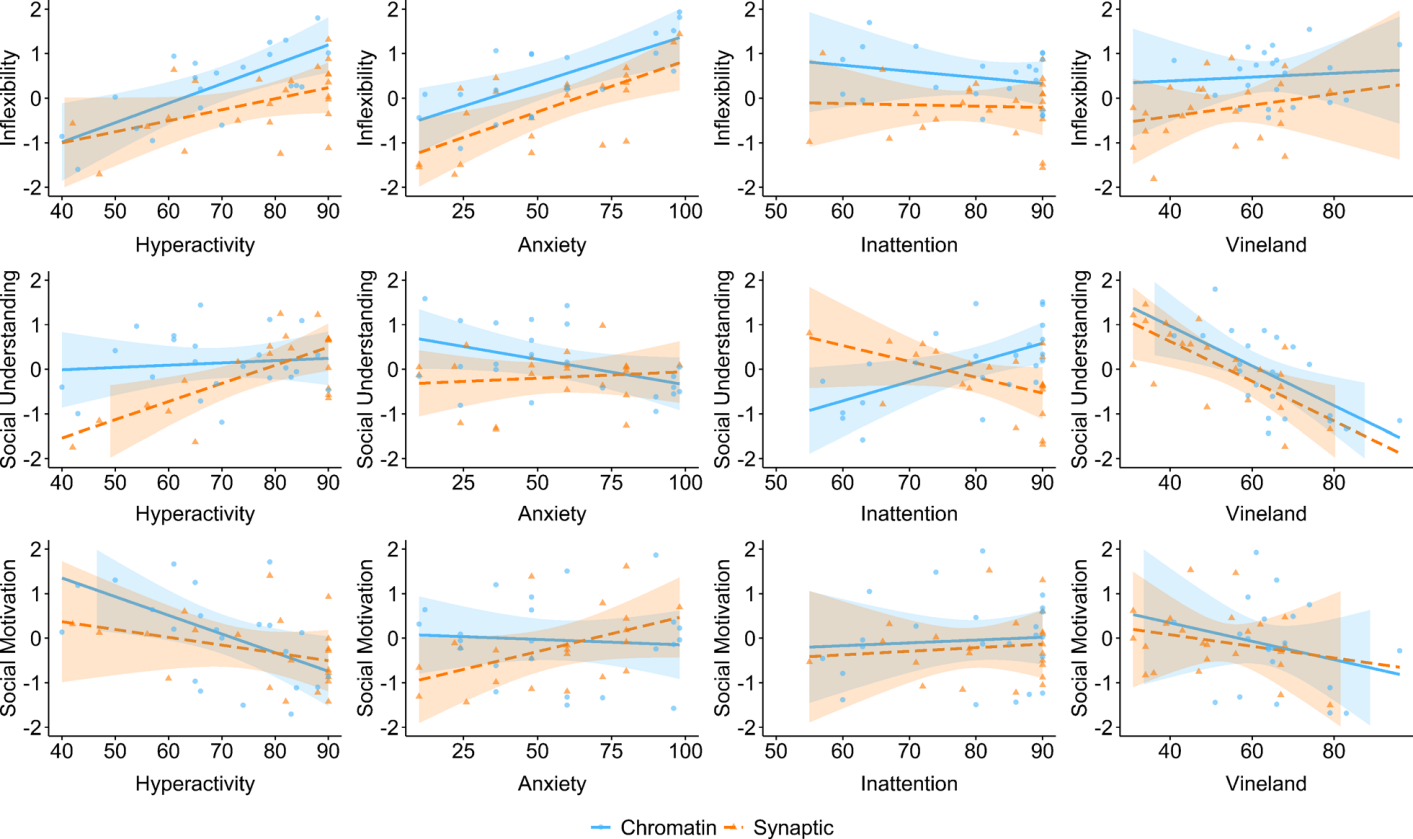
Relationships between ASC and non-ASC behavioural characteristics, within each Functional Network Group. The fitted lines are ASC PCA factor scores predicted by behavioural standardised coefficients, for each FNG (blue = chromatin, orange = synaptic). Individual data points (orange triangles = synaptic; blue circles = chromatin) are partial residuals, i.e. observed data accounting for all the variables included in the models (age, gender, FNG, general ability, and interactions with behavioural measures). The shaded areas, respectively, in blue and orange for each FNG, are confidence intervals at 95%

Multi-model inference indicated that interactions between FNG and non-ASC behavioural factors contributed to the set of candidate models, suggesting that there may be different predictors of each dimension within functional network groups. To inspect these relationships, we plotted linear models for each ASC dimension and non-ASC variables of interest. Figure 3 illustrates associations between ASC components and behavioural predictors within both functional network groups. The positive effect of global adaptive function on Social Understanding was the same across both groups, and no group-specific effect of global adaptive function was observed for either Inflexibility or Social Motivation. We observed an interaction between group, hyperactivity, and Inflexibility, whereby the association between hyperactivity and Inflexibility is more pronounced within the chromatin group (whereas the association between anxiety and Inflexibility is constant across groups). We also observed group-specific predictors of impaired Social Understanding (anxiety and inattention for the chromatin group, hyperactivity for the synaptic group). For Social Motivation, contrasting relationships were observed within groups: hyperactivity predicts social disinhibition within the chromatin group, whereas anxiety predicts social withdrawal within the synaptic group. For effect sizes of each coefficient and predictor, see Additional file 1: Table S7. In summary, we found evidence for some shared and some distinct predictors of ASC dimensions across FNGs.

## Discussion

Numerous data-driven analyses have implicated discrete functional networks such as chromatin regulation, synaptic communication, and cytoskeletal architecture in the neuronal origins of ASD [24]. However, to date there has been no evidence that pathogenic variants within these gene sets influence the prevalence or types of autistic characteristics amongst individuals with ID. In this exploratory study, we address this gap in the literature directly. The overall likelihood of autism-relevant characteristics was high across the sample and did not differ between groups. Therefore, we find no evidence that disruption to synaptic physiology leads to overall higher risk of autism than disruption to chromatin regulation, or vice versa. However, after separating autism-relevant questionnaire items into dimensions, and taking into account background variables, we found that gene functional networks predicted specific aspects of autism phenotype and predicted co-occurrence between ASC and other behavioural characteristics. Thus we find preliminary support for a model whereby genetic diagnoses converging on similar neuronal functions can influence dimensions of social development via shared, specific mechanisms.

### Disorders of chromatin regulation, inflexibility, and cognitive control

Our within-sample modelling found that disorders of chromatin regulation are associated with elevated Inflexibility. These specific behavioural characteristics can have important knock-on consequences for individuals’ access to educational and psychosocial interventions and exert strong influence on family life and well-being. Inflexibility may be masked by more overt difficulties, for example communication impairments or oppositional behaviour. An important next step is to consider how chromatin regulation is related to Inflexibility, at the levels of cognitive development, neural systems, and molecular neurobiology. The observed relationship between hyperactivity and inflexibility within the chromatin group suggests disproportionate impact of chromatin dysregulation on cognitive control systems. We also found that hyperactivity and inattention predicted social disinhibition and social understanding, uniquely within the chromatin group, further supporting the potential importance of cognitive control for social development of these individuals. At a neural level, cognitive control relies upon functional integration between multiple cortical areas. Chromatin-associated genes could influence functional integration via early development of relevant cortical and subcortical structures, later white matter development, or dynamic remodelling of neural networks [25, 26].

### Disorders of synaptic physiology and social–emotional development

Individuals within the synaptic group had more severe ID on average, which did not translate to higher SRS total scores or simple differences in ASC factor scores, emphasising that autism characteristics are not an inevitable consequence of global cognitive impairments. The predictors of ASC dimensions within the synaptic group contrast with those observed within the chromatin group, suggesting that a distinct set of developmental mechanisms may contribute to the social–emotional difficulties of the synaptic group. Within this group (only), we observed that anxiety and social withdrawal are correlated, and hyperactivity and social understanding are negatively linked. Further investigation is warranted to determine whether these associations highlight specific relationships between synaptic physiology, motor control, emotional arousal, and social interaction or are common associations amongst individuals with severe ID. For the Social Motivation dimension, higher rates of residual deviance and lower model weights indicate that there are unmeasured predictors contributing to variability in this heterogeneous component. Further research is required to disentangle the factors contributing to social withdrawal versus social disinhibition, both of which can be distressing and impairing for the individual and their social circle.

## Limitations

The functional networks approach is advantageous in identifying broad group-based associations and spotlighting potential mechanistic convergence; however, we openly acknowledge that the approach will mask potentially important gene-specific characteristics. Our approach of allocating genes to network groups is based on integration of multiple literature sources, each limited by existing functional data. Boundaries between networks are difficult to define; for example, chromatin-associated genes will have downstream effects on synaptogenesis, neurotransmission, and plasticity, by regulating expression of synaptic-relevant targets [27–29]. We included components of the Wnt signalling pathway (*DDX3X* and *CTNNB1*) in the “synaptic” group, because of emerging evidence that Wnt signalling directly “tunes” neurotransmitter release and modulates synaptic plasticity [30]. Similarly, we included *DYRK1a* in the synaptic group because there are multiple lines of experimental evidence supporting a direct role for this kinase in regulation of presynaptic vesicle cycling [31]. Ultimately, both data-driven and experimental approaches to functional network definitions would avoid bias in group allocations. In larger studies, it would be advantageous to apply permutation-based bootstrapping methods to assess the stability of models with variable group memberships.

Several further limitations are recognised. First, the sample size was small, and we acknowledge the potential for type I or type II error. The study was intended to be exploratory, and future pre-registered, multi-site studies with larger samples are needed to test the stability of our three-component solution, explore a wider range of potential predictors (e.g. epilepsy, motor deficits, sensory impairments), and determine the robustness of the functional networks phenotyping approach and our specific findings. Larger samples would allow parallel PCA to determine whether ASC structure is constant across FNGs. The overt focus of the study on social and emotional characteristics may have biased recruitment of the sample towards individuals presenting with difficulties in these areas. While our study design and inclusion criteria did not specify severity of neurodevelopmental difficulties, and methods were selected to be accessible for a wide spectrum, measures may not be equivalently sensitive to strengths and difficulties for individuals with either very severe or very mild ID. Indeed, there is substantial evidence that SRS item responses and scaled scores are influenced by several background factors, namely age, expressive language function, non-verbal IQ and behaviour problems [32]. These factors vary within our study population and could thus confound the observed dimensional structure and modelling results. This study deployed carer-report questionnaire measures only, and future studies could obtain richer insights via multi-informant reports, interview schedules, observational methods, and neuropsychological assessments. Related to this, using measures specifically designed for and validated in ID populations could improve sensitivity and precision when measuring ASC and other dimensions. ASC are expected to change with chronological and developmental age, perhaps in a gender-modified fashion [33], necessitating longitudinal studies. Lastly, socioeconomic status and family characteristics such as household structure, parental education, family stress, and parental mental health may also interact with ASC, with complex bidirectional relationships between child and family factors [34], which may also encompass genetic diagnosis [3].

## Conclusions

In this study, the genetic cause of an individual’s ID (classified by functional network) did not predict overall likelihood of autistic features, but did influence dimensional autism characteristics and co-occurrences. These results indicate that genetic diagnosis can be clinically relevant to understanding the social abilities and difficulties of individuals with ID, but only if genetic diagnosis is considered in the context of a multi-faceted assessment, encompassing dimensions within and beyond the autism spectrum. Chromatin regulator variants were associated with elevated Inflexibility, suggesting disproportionate impact on neural systems underlying cognitive control. Furthermore, we report early insights into multiple pathways contributing to Social Understanding and Social Motivation, which may be differentially influenced by gene functional network groups. These data highlight the diversity of social and emotional characteristics that contribute to autism in the context of ID and corresponding diversity of genetic and neurodevelopmental mechanisms. It is not yet known whether valuable outcomes such as social inclusion and mental health could be improved if these underlying mechanisms were to be considered targets for intervention. Future research should seek to replicate and extend these findings and investigate the molecular, neural, cognitive, and interpersonal mechanisms contributing to the emergent tapestry of social function for individuals with ID and their families.

## Supplementary information

**Additional file 1:** Supplementary tables.

## Data Availability

The data that support the findings of this study are available to other ethically approved research projects from the corresponding author, KB.

## Abbreviations

AIC: Akaike information criterion
ASC: Autism spectrum characteristics
ASD: Autism spectrum disorder
CPRS: Conners Parent Rating Scales
DBC: Developmental Behaviour Checklist
FNG: Functional networks groups
ID: Intellectual disability
IQ: Intelligence quotient
PCA: Principal components analysis
SRS: Social Responsiveness Scale, Second edition
