# Supplementary material for: Gene functional networks and autism spectrum characteristics in young people with intellectual disability: a dimensional phenotyping study
Source: Mol Autism. 2020 Dec 11;11:98. doi: 10.1186/s13229-020-00403-9 (PMC7731560; doi:10.1186/s13229-020-00403-9)
Supplement: Supplementary file 1 — Additional file 1: Supplementary tables. [file 13229_2020_403_MOESM1_ESM.pdf]

Additional file 1

Page 2	Table S1	Evidence for functional network classification of chromatin-associated and synaptic-associated Intellectual Disability genes
Page 5	Table S2	Participant numbers by gene and functional network group
Page 6	Figure S1	Scree plots, extraction and rotation values for Principal Components Analysis
Page 8	Table S3	Rotated component matrix for three-component solution
Page 10	Table S4	PCA solution with oblique rotation
Page 12	Table S5	Pearson correlation matrix of component scores (Orthogonal and Oblique rotations)
Page 13	Table S6	Top-ranked models with ΔAIC <2, for each ASC dimension
Page 15	Table S7	Effect size tables of three-top ranked models for each ASC dimension

Table S1. Evidence for functional network classification of chromatin-associated and synaptic-associated Intellectual Disability genes

Gene	Protein	Biochemical and cellular function	Human adult brain expression	Human developmental brain expression	Synaptic proteome	Synaptic-relevant GO Biological processes	Chromatin-relevant GO Biological processes
Resource	ncbi.nlm.nih.gov/omim	genecards.org	braineac.org	hbatlas.org	SynaptomeDB.org	http://amigo.geneontology.org/	http://amigo.geneontology.org/
CHROMATIN GROUP							
ARID1B	At-Rich Interaction Domain-Containing Protein 1b	Component of SWI/SNF chromatin remodeling complexes, changing chromatin structure by altering DNA-histone contacts within a nucleosome in an ATP-dependent manner	Max – cerebellum<br>Min - thalamus	Peaks day 150, stable postnatal	NO	• Differentiation of interneurons<br>• excitatory / inhibitory balance<br>• Dendritic morphology	• SWI/SNF complex<br>• chromatin-mediated maintenance of transcription
EHMT1	Euchromatic Histone Methyltransferase 1	Histone methyltransferase that specifically mono- and dimethylates 'Lys-9' of histone H3 (H3K9me1 and H3K9me2, respectively) in euchromatin	Max – white matter<br>Ubiq in cortex	Peaks early gestation, declines through prenatal, stable postnatal	NO	Nil	• DNA methylation<br>• chromatin organization
KAT6B	Lysine Acetyltransferase 6b	Histone acetyltransferase which may be involved in both positive and negative regulation of transcription. Required for RUNX2-dependent transcriptional activation	Not in database	Peaks day 100, stable postnatal	NO	Nil	• negative regulation of transcription, DNA-templated<br>• positive regulation of transcription by RNA polymerase II<br>• nucleosome assembly
SMARCA2	Swi/Snf-Related, Matrix-Associated, Actin-Dependent Regulator Of Chromatin, Subfamily A, Member 2	Component of SWI/SNF chromatin remodeling complexes that carry out key enzymatic activities, changing chromatin structure by altering	Max – cortex<br>Min – white matter	Increases during prenatal life. Stable postnatal	NO	Nil	

		DNA-histone contacts within a nucleosome in an ATP-dependent manner.					
SETD5	Set Domain-Containing Protein 5	Displays histone methyltransferase activity and monomethylates 'Lys-9' of histone H3 in vitro. Probable transcriptional regulator that acts via the formation of large multiprotein complexes that modify and/or remodel the chromatin.	Max – cerebellum<br>Min - medulla	Peaks early gestation, steady decline	NO	Nil	• covalent chromatin modification<br>• regulation of chromatin organization
**SYNAPTIC GROUP**							
CTNNB1	Catenin, Beta-1	Downstream component of the canonical Wnt signalling pathway	Max – cerebellum, thalamus<br>Min – putamen, SNIG	Peaks early gestation, then steady	YES	• synaptic vesicle transport<br>• synaptic vesicle clustering<br>• synaptic transmission<br>• Wnt signalling pathway, calcium modulating pathway	• Positive regulation of transcription
DDX3X	Dead/H Box 3, X-Linked	Multifunctional ATP-dependent RNA helicase.	Ubiq	Peaks early gestation, then steady	YES	• Wnt signalling pathway	• DNA helicase activity<br>• Translational and transcriptional regulation<br>• Positive regulation of gene expression<br>• RNA secondary structure unwinding
DLG3	Discs, Large Homolog 3	Membrane-associated guanylate kinase	Max – hippocampus, cortex<br>Min – medulla, white matter	Increases across prenatal, declines from late childhood	YES	• structural constituent of postsynaptic density<br>• regulation of postsynaptic membrane neurotransmitter receptor levels<br>• maintenance of postsynaptic density structure<br>• regulation of NMDA receptor activity	Nil

PAK3	p21 protein (Cdc42/Rac)-Activated Kinase 3	Serine/threonine protein kinase. Acts as downstream effector of small GTPases	Max – hippocampus, cortex<br>Min – cerebellum, white matter	Increases during prenatal life. Stable postnatal	NO (but PAK1 yes)	• synapse organization<br>• dendritic spine morphogenesis	Nil
SHANK3	Sh3 And Multiple Ankyrin Repeat Domains 3	Major scaffold postsynaptic density protein	Max – hippocampus, putamen<br>Min – white matter, medulla	Not in database (SHANKS 1 and 2 – increases across prenatal, stable / declines postnatal)	YES	• synapse assembly<br>• positive regulation of long-term neuronal synaptic plasticity<br>• positive regulation of synaptic transmission, glutamatergic<br>• AMPA and NMDA glutamate receptor clustering	Nil
STXBP1	Syntaxin-Binding Protein 1	Regulation of synaptic vesicle docking and fusion through interaction with GTP-binding proteins	Max – cortex<br>Min – white matter	Increases during prenatal life. Stable postnatal	YES	• vesicle docking involved in exocytosis<br>• regulation of synaptic vesicle priming<br>• negative regulation of synaptic transmission, GABAergic<br>• positive regulation of calcium ion-dependent exocytosis<br>• long-term synaptic depression	Nil
TRIO	Triple Functional Domain	Guanine nucleotide exchange factor (GEF) for RHOA and RAC1 GTPases	Max – cerebellum, cortex<br>Min – white matter	Peaks day 150, declines postnatal	YES	• regulation of Rho protein signal transduction	Nil
ZDHHC9	Zinc Finger Dhhc Domain-Containing Protein 9	Palmitoyltransferase	Max – white matter<br>Min - cerebellum	Peaks day 100, stable postnatal (adolescent increase?)	NO	• Protein targeting to membrane	Nil
DYRK1A	Dual-Specificity Tyrosine Phosphorylation-Regulated Kinase 1a	Dual-specificity kinase which possesses both serine/threonine and tyrosine kinase activities. Modulates alternative splicing by phosphorylating the splice factor SRSF6	Max – cerebellum<br>Min – white matter	Peaks early gestation, declines through prenatal, stable postnatal	NO	• Protein tyrosine kinase	• negative regulation of mRNA splicing, via spliceosome<br>• transcription coactivator activity<br>• positive regulation of transcription, DNA-templated

Table S2. Participant numbers by gene and functional network group

Chromatin		Synaptic	
**Gene**	**N**	**Gene**	**N**
ARID1B	6	*CASK*	1
EHMT1	7	*CTNNB1*	1
KAT6B	1	*DDX3X*	9
SETD5	8	*DLG3*	2
SMARCA2	1	*DYRK1A*	2
		PAK3	1
		SHANK3	3
		STXBP1	8
		TRIO	1
		ZDHHC9	1

Figure S1. Scree plots, extraction and rotation values for Principal Components Analysis

<u>Preliminary PCA on all SRS-2 items</u>

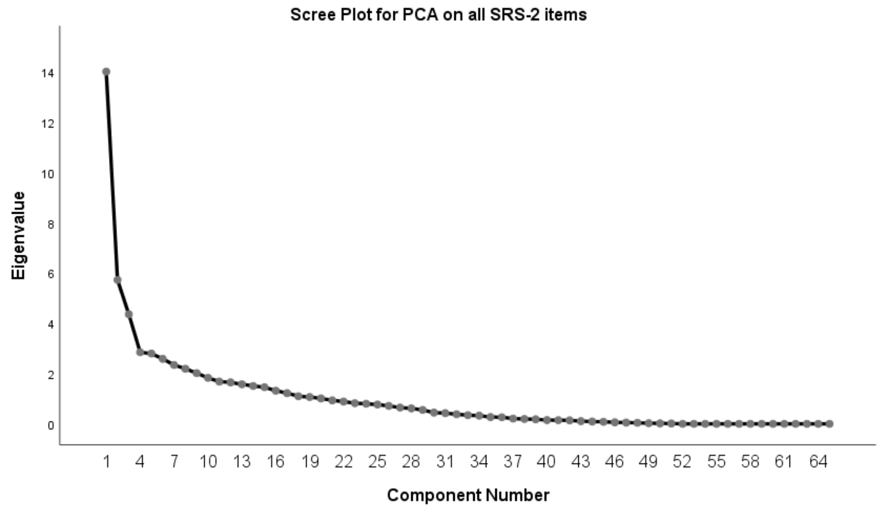

		Component				
		1	2	3	4	5
Extraction sums of squared loadings	Eigenvalue	14.008	5.726	4.358	2.848	2.796
	% of variance	21.551	8.810	6.704	4.382	4.302
	Cumulative %	21.551	30.361	37.065	41.447	45.749

Extracted values for the first five components produced by initial PCA, performed on all 65 SRS-2 items.

Final PCA on reduced number of SRS-2 items (30 items)

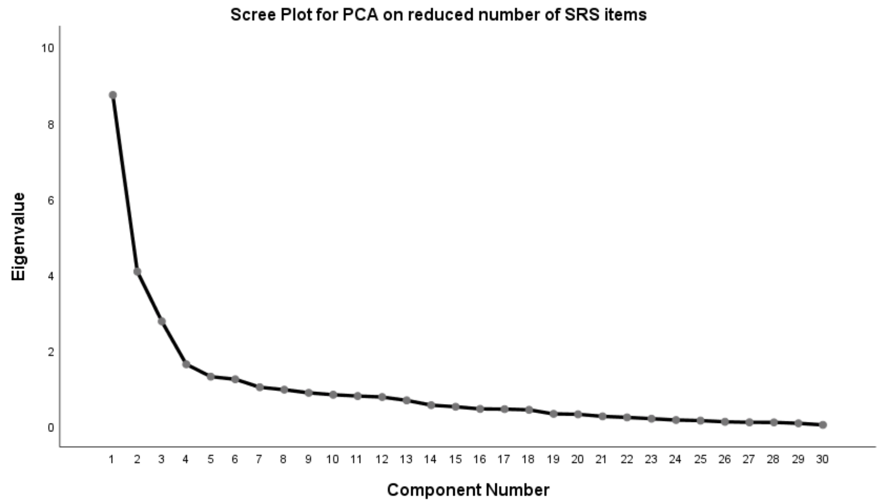

		Component		
		1	2	3
Extraction sums of squared loadings	Eigenvalue	8.729	4.072	2.760
	% of variance	29.095	13.574	9.200
	Cumulative %	29.095	42.669	51.869
Rotation sums of squared loadings	Eigenvalue	6.786	5.908	2.867
	% of variance	22.618	19.694	9.556
	Cumulative %	22.618	42.312	51.869

Extracted and rotated values for the three component solution produced by a second PCA, performed on a reduced number of SRS-2 items (30)

Table S3. Rotated component matrix for three-component solution

Item	Component		
	Inflexibility	Social Understanding	Social Motivation
Difficulty with changes to routine	**.777**	.053	.060
Overwhelmed in situations with lots going on	**.776**	-.077	.149
Sensory sensitivity	**.752**	-.112	-.058
Has fixated patterns of thought	**.732**	.092	.258
Tense in social situations	**.731**	-.032	.179
Inflexible	**.697**	.172	.158
When stressed shows rigid behaviours	**.689**	.150	.017
Too literal	**.653**	.318	.127
Stares into space	**.634**	.183	-.073
Behaves in ways that are strange or bizarre	**.555**	**.516**	-.198
Has difficulty relating to peers	**.530**	.371	-.065
Repetitive behaviours	**.451**	**.406**	**-.419**
Aware when being too loud	-.093	**.706**	.046
Aware of others’ thoughts and feelings	.125	**.695**	-.016
Knows when standing too close to others	-.102	**.693**	.003
Offers comfort to others when they are sad	-.048	**.686**	.258
Understands cause and effect	.189	**.675**	-.204
Recognises when something is unfair	-.076	**.658**	.156
Understands the meaning of others’ tone	.118	**.613**	.227
Regarded by others as odd	.292	**.598**	-.060
Awkward in turn-taking interactions	.289	**.572**	.042
Socially awkward	**.443**	**.550**	-.096
Shows unusual sensory interests	**.447**	**.548**	-.178
Difficulty communicating thoughts	.209	**.542**	**.431**

**Item**	**Component**		
	**Inflexibility**	**Social Understanding**	**Social Motivation**
Walks between people	.101	**.505**	-.301
Avoids initiating social interactions	.220	-.091	**.753**
Poo self-confidence	-.007	.219	**.700**
Avoids emotional closeness with others	.399	.128	**.601**
Silly	**.462**	.129	**-.523**
Gets frustrated trying to communicate ideas	**.479**	-.008	**.518**

Variance explained: 51.87%. Extraction method: Principal Components with Varimax rotation and Kaiser normalization. Item loadings > 0.4 are in bold font.

Table S4. PCA solution with oblique rotation

Pattern matrix for three-component solution with oblique rotation

Item	Component		
	Inflexibility	Social Understanding	Social Motivation
Overwhelmed in situations with lots going on	**.839**	-.231	.136
Sensory sensitivity	**.820**	-.279	-.073
Difficulty with changes to routine	**.813**	-.100	.049
Tense in social situations	**.782**	-.172	.168
Has fixated patterns of thought	**.758**	-.035	.250
Inflexible	**.703**	.050	.152
When stressed shows rigid behaviours	**.698**	.018	.152
Stares into space	**.633**	.058	-.079
Too literal	**.627**	.212	.125
Has difficulty relating to peers	**.484**	.279	-.064
Behaves in ways that are strange or bizarre	**.479**	**.418**	-.195
Aware when being too loud	-.245	**.772**	.065
Offers comfort to others when they are sad	-.192	**.757**	.276
Knows when standing too close to others	-.252	**.757**	.021
Recognises when something is unfair	-.216	**.726**	.174
Aware of others' thoughts and feelings	-.013	**.711**	-.001
Understands cause and effect	.059	**.662**	-.192
Understands the meaning of others' tone	-.001	**.643**	.240
Regarded by others as odd	.185	**.570**	-.050
Difficulty communicating thoughts	.110	**.565**	**.443**
Awkward in turn-taking interactions	.188	**.550**	.051
Walks between people	.000	**.493**	-.301
Socially awkward	.354	**.485**	-.090

Item	Component		
	Inflexibility	Social Understanding	Social Motivation
Shows unusual sensory interests	.359	**.476**	-.172
Avoids initiating social interactions	.256	-.086	**.750**
Poor self-confidence	-.050	.286	**.709**
Avoids emotional closeness with others	.399	.097	**.600**
Silly	**.460**	.003	**-.529**
Gets frustrated trying to communicate ideas	**.512**	-.069	**.512**
Repetitive behaviours	.391	.307	**-.418**

Extraction method: Principal Components with Promax rotation and Kaiser normalization. Item loadings > 0.4 are in bold font.

Table S5. Pearson correlation matrix of component scores (Orthogonal and Oblique rotations)

	**Inflexibility (Orthogonal)**		**Social Understanding (Orthogonal)**		**Social Motivation (Orthogonal)**		**Inflexibility (Oblique)**		**Social Understanding (Oblique)**		**Social Motivation (Oblique)**	
**N=52**	*r*	*p*	*r*	*p*	*r*	*p*	*r*	*p*	*r*	*p*	*r*	*p*
**Inflexibility (Orthogonal)**			.000	1.000	.000	1.000	.982	<.001	.193	.171	-.020	.890
**Social Understanding (Orthogonal)**	.000	1.000			.000	1.000	.191	.176	.981	<.001	-.073	.606
**Social Motivation (Orthogonal)**	.000	1.000	.000	1.000			.011	.941	-.020	.886	.997	<.001
**Inflexibility (Oblique)**	.982	<.001	.191	.176	.011	.941			.376	.006	-.023	.873
**Social Understanding (Oblique)**	.193	.171	.981	<.001	-.020	.886	.376	.006			-.096	.499
**Social Motivation (Oblique)**	-.020	.890	-.073	.606	.997	<.011	-.023	.873	-.096	.499		

Table S6. Complete table of top-ranked models with ΔAIC <2, for each ASC dimension

Component	Models	N Variables	AIC Weight	AICc	ΔAIC	Residual Deviance	Explained Deviance or D squared
Inflexibility	Anxiety + FNG + Hyperactivity + Vineland	4	0.221	100.21	0	17.81	0.623
	Anxiety + FNG + Hyperactivity	3	0.202	100.39	0.18	18.89	0.598
	Anxiety + FNG + Hyperactivity + FNG x Hyperactivity	5	0.175	100.68	0.466	18	0.619
	Anxiety + FNG + Hyperactivity + Vineland + FNG x Hyperactivity	7	0.136	101.19	0.973	17.1	0.638
	Anxiety + FNG + Hyperactivity + Vineland + FNG x Vineland	7	0.09	102.01	1.791	17.41	0.631
	Age + Anxiety + FNG + Hyperactivity	4	0.099	102.01	1.799	18.54	0.607
	Anxiety + FNG + Hyperactivity + Inattention	5	0.096	102.06	1.847	18.56	0.607
Social Understanding	Anxiety + FNG + Hyperactivity + Inattention + Vineland + FNG x Hyperactivity + FNG x Inattention	9	0.174	114.93	0	20.26	0.5952544
	FNG + Hyperactivity + Inattention + Vineland + FNG x Hyperactivity + FNG x Inattention	8	0.133	115.47	0.537	21.98	0.5607631
	Anxiety + FNG + Gender + Hyperactivity + Inattention + Vineland + FNG x Hyperactivity + FNG x Inattention	10	0.131	115.49	0.564	19.05	0.6193666
	Anxiety + FNG + Hyperactivity + Inattention + Vineland + Anxiety x FNG + FNG x Hyperactivity + FNG x Inattention	10	0.106	115.93	0.998	19.24	0.6156723

Component	Models	N Variables	AIC Weight	AICc	ΔAIC	Residual Deviance	Explained Deviance or D squared
Social Understanding	FNG + Gender + Hyperactivity + Inattention + Vineland + FNG x Hyperactivity + FNG x Inattention	9	0.1	116.04	1.107	20.76	0.5851734
	Age + FNG + Gender + Hyperactivity + Inattention + Vineland + FNG x Hyperactivity + FNG x Inattention	10	0.1	116.04	1.112	19.28	0.614705
	Anxiety + FNG + Gender + Hyperactivity + Inattention + Vineland + Anxiety x FNG + FNG x Hyperactivity + FNG x Inattention	11	0.097	116.1	1.168	17.85	0.6433202
	Age + Gender + Hyperactivity + Vineland	6	0.088	116.29	1.361	25.46	0.4912235
	Age + FNG + Hyperactivity + Inattention + Vineland + FNG x Hyperactivity + FNG x Inattention	9	0.07	116.76	1.835	21.1	0.5784112
Social Motivation	Hyperactivity + Inattention	4	0.34	127.12	0	36.37	0.154765
	Hyperactivity	3	0.328	127.19	0.071	38.43	0.1067548
	Age + Hyperactivity	4	0.199	128.19	1.072	37.24	0.1343813
	Age + Hyperactivity + Inattention	5	0.133	128.99	1.871	35.83	0.1672165

N variables = number of parameters for each model, AIC weight= is the probability of each model of being the best model, or relative evidence for each model. AICc=aic criterion of model selection, corrected for smaller sample size, δAIC=AIC difference between the best fitting model (equal to zero) and the second best one. residual deviance=distance between the data and the model. FNG=functional network group refers to the synaptic/chromatin grouping.

Table S7. Effect size tables of three-top ranked models for each ASC dimension

The following tables describe for each ASC dimension (Inflexibility, Social Understanding and Social Motivation) the confidence estimates and predictors included in the three top ranked models reported in the main text (Table 2).

Inflexibility

	**First Model**			**Second Model**			**Third Model**		
Coefficient	*Estimates*	*Conf. Int (95%)*	*P-Value*	*Estimates*	*Conf. Int (95%)*	*P-Value*	*Estimates*	*Conf. Int (95%)*	*P-Value*
Intercept	-4.00 ***	-5.87 – -2.13	**<0.001**	-2.77 ***	-3.84 – -1.69	**<0.001**	-3.55 ***	-5.05 – -2.04	**<0.001**
Anxiety	0.02 ***	0.01 – 0.03	**<0.001**	0.02 ***	0.01 – 0.03	**<0.001**	0.02 ***	0.01 – 0.03	**<0.001**
FNG	-0.54 *	-1.01 – -0.08	**0.024**	-0.71 **	-1.14 – -0.29	**0.002**	0.78	-1.30 – 2.86	0.455
Hyperactivity	0.03 ***	0.02 – 0.05	**<0.001**	0.03 ***	0.01 – 0.04	**<0.001**	0.04 ***	0.02 – 0.06	**0.001**
Vineland	0.01	-0.00 – 0.03	0.114						
FNG x Hyperactivity							-0.02	-0.05 – 0.01	0.148
Observations	45			45			45		
R^2 Nagelkerke	0.739			0.718			0.736		

** p<0.05 ** p<0.01 *** p<0.001*

Social Understanding

	First Model			Second Model			Third Model		
Predictors	*Estimates*	*Conf. Int (95%)*	*P-Value*	*Estimates*	*Conf. Int (95%)*	*P-Value*	*Estimates*	*Conf. Int (95%)*	*P-Value*
Intercept	-3.39 *	-6.41 – -0.38	**0.028**	-3.71 *	-6.78 – -0.63	**0.019**	-4.10 *	-7.22 – -0.99	**0.011**
Anxiety	-0.01	-0.02 – 0.00	0.084				-0.01	-0.02 – 0.00	0.081
FNG	3.34	-0.09 – 6.77	0.056	3.84 *	0.36 – 7.31	**0.031**	3.62 *	0.22 – 7.02	**0.037**
Hyperactivity	0.01	-0.02 – 0.03	0.649	0.00	-0.02 – 0.03	0.774	0.00	-0.02 – 0.03	0.741
Inattention	0.06 ***	0.03 – 0.09	**0.001**	0.06 ***	0.03 – 0.09	**0.001**	0.06 ***	0.03 – 0.10	**<0.001**
Vineland	-0.02 *	-0.04 – -0.00	**0.027**	-0.02 *	-0.04 – -0.00	**0.023**	-0.02 *	-0.04 – -0.00	**0.020**
FNG x Hyperactivity	0.04 *	0.00 – 0.08	**0.032**	0.04 *	0.01 – 0.08	**0.022**	0.04 *	0.00 – 0.08	**0.032**
FNG x Inattention	-0.08 **	-0.13 – -0.03	**0.002**	-0.09 ***	-0.14 – -0.04	**0.001**	-0.08 **	-0.13 – -0.03	**0.001**
Gender							0.35	-0.12 – 0.83	0.140
Observations		45			45			45	
R^2 Nagelkerke		0.721			0.691			0.742	

* *p<0.05* ** *p<0.01* *** *p<0.001*

Social Motivation

	First Model			Second Model			Third Model		
Predictors	*Estimates*	*Conf. Int (95%)*	*P-Value*	*Estimates*	*Conf. Int (95%)*	*P-Value*	*Estimates*	*Conf. Int (95%)*	*P-Value*
Intercept	0.28	-1.81 – 2.37	0.787	1.48 *	0.06 – 2.89	**0.042**	1.92 *	0.31 – 3.54	**0.021**
Hyperactivity	-0.03 **	-0.05 – -0.01	**0.008**	-0.02 *	-0.04 – -0.00	**0.028**	-0.02 *	-0.04 – -0.00	**0.031**
Inattention	0.02	-0.01 – 0.05	0.130						
Age							-0.00	-0.01 – 0.00	0.253
Observations	45			45			45		
R^2 Nagelkerke	0.223			0.158			0.196		

* *p<0.05* ** *p<0.01* *** *p<0.001*

Legend: FNG= Functional Network Group; R^2 Nagelkerke= pseudo R-squared that ranges between 0-1 and represents a scoring and logarithmic rule that evaluates the overall performance of the model or how well the model explains the data.
